# Structures and strategies for retaining an international pediatric cohort from birth: Lessons from The Environmental Determinants of Diabetes in the Young (TEDDY) study

**DOI:** 10.1016/j.conctc.2024.101405

**Published:** 2025-01-23

**Authors:** Patricia Gesualdo, Jessica Melin, Rachel Karban, Claire Crouch, Michael Killian, Diane Hopkins, Annika Adamsson, Joanna Stock, Suzanne Bennett Johnson, Judith Baxter, TEDDY Study Group

**Affiliations:** a Barbara Davis Center for Diabetes, University of Colorado, Aurora, CO, USA; b Department of Clinical Sciences Malmö, Lund University, Sweden; c Pacific Northwest Research Institute, Seattle, WA, USA; d Center for Biotechnology and Genomic Medicine, Medical College of Georgia, Georgia Regents University, Augusta, GA, USA; e Institute of Biomedicine, Research Centre for Integrative Physiology and Pharmacology, and Centre for Population Health Research, University of Turku, And Department of Pediatrics, Turku University Hospital, Turku, Finland; f Institute of Diabetes Research, Helmholtz Zentrum München, And Klinikum Rechts der Isar, Technische Universität München, And Forschergruppe Diabetes e.V., Neuherberg, Germany; g Department of Behavioral Sciences and Social Medicine, Florida State University College of Medicine, Tallahassee, FL, USA

**Keywords:** Retention, Longitudinal study, Type 1 diabetes, Pediatric, Strategies

## Abstract

**Background:**

Retention of study participants in observational studies is essential to maintaining the representativeness of the population, minimizing selection bias, and assuring sufficient statistical power. The aim of this report is to describe the structures and strategies used to retain participants in The Environmental Determinants of Diabetes in the Young (TEDDY) Study, an observational study of children at increased genetic risk for type 1 diabetes followed in an intensive protocol from birth until age 15.

**Methods:**

Teague et al.’s systematic review of study retention strategies identified four domains: barrier reduction; community building; follow-up/reminder; and tracing strategies (1). TEDDY retention strategies were categorized into each of these domains. A fifth category presented strategies unique to TEDDY.

**Results:**

TEDDY employed over one hundred retention strategies during the 15 years of follow-up; many could be categorized within the Teague domains. Strategies unique to TEDDY included (1) study structures to support retention; (2) risk communication and education strategies specific to this population; (3) Data-informed retention strategies that addressed protocol challenges in real-time; and (4) implementation of a re-engagement protocol for those who had withdrawn from the study.

**Conclusion:**

Pediatric cohort studies should include strategies, structures, and resources to address retention at the study's initiation and on an ongoing basis. Retention strategies should not remain static but change with the developmental needs of the child. Collecting and analyzing data on an ongoing basis permits retention strategies to be put in place to address protocol and retention challenges in real time.

**Trial registration:**

ClinicalTrials.gov Identifier: NCT00279318.

## 1 Introduction

Longitudinal cohort studies have the advantage of assessing time-varying relationships between exposures collected over time and the progression of a disease in the absence of treatment [2]. However, attrition can threaten the validity of any longitudinal study that seeks to understand the natural history of disease [3]. The loss to follow-up can lower the ability of the study to detect true associations existing in a population and missing data during follow-up may lead to incorrect statistical inference and conclusions concerning the relationships between exposures and health outcomes. Participant retention can be challenging, especially in pediatric observational cohort studies where there is no treatment, the study duration is long, and protocols require time and effort from the child and parent. Participant motivation to remain in a study may weaken over time [4]. Recommended strategies to promote study retention include using experienced study coordinators, consistency of staff over time, changing strategies depending on the phase of the study, building rapport with families, tailoring strategies to the individual needs of the participant, and engaging the child with communication strategies, activities and incentives that are age-appropriate and change as the child matures [[4], [5], [6], [7], [8], [9]].

The Environmental Determinants of Diabetes in the Young (TEDDY) Study is an international prospective cohort study designed to identify environmental factors and gene-environment interactions that may trigger type 1 diabetes in genetically at-risk children [10]. The study protocol, designed with an intense follow up of frequent visits and various forms of data collection, presented retention challenges. As the TEDDY Study reaches the 20th year since the initial enrollment began, this milestone provided an opportunity to review the successes of retaining this unique pediatric cohort from birth to 15 years of age. This report describes structures and strategies used to retain study participants and highlights innovative strategies developed over the course of the TEDDY study.

## 2 Materials and methods

### 2.1 The TEDDY Study

Details of the TEDDY Study design, protocol and follow-up schedule have been previously published [11,12]. Briefly, infants identified with increased genetic risk for type 1 diabetes were enrolled at six centers in Finland, Germany, Sweden, and the United States between July 2004 and February 2010. Children were followed until the development of type 1 diabetes or 15 years of age. Of the 424,788 infants screened at birth, 21,589 were eligible for TEDDY and 8667 (40 %) enrolled. Children were followed every 3 months until 4 years of age, with study visits every six months thereafter. However, children who developed type 1 diabetes-related autoantibodies continued with every 3-month study visits. Study visits lasted 1–2 h and included a blood draw, clinical measurements, interviews, and questionnaires (Table 1). The study was approved by local institutional review or ethics boards and was monitored by an External Evaluation Committee formed by the National Institutes of Health (NIH).

**Table 1:** Data collected in the TEDDY study.

Data Collected	Frequency by Age
Blood	Every clinic visit^a^
Stool	Monthly up to 4 years of age; four times a year up to 10 years of age
Tap water	Child-age 9 months; every two years after 3 years of age
Toenails	Child-age 2 years; annually thereafter
Nasal swab	Every clinic visit
Urine	Every clinic visit
Activity Meter	Child-age 5 years; annually up to 10 years of age
Weight and Height	Every clinic visit
Three-day food record	Every 3 months up to 1 year; biannually up to 10 years of age
Parent Questionnaires	Child-age 3, 6, 15, and 27 months; annually thereafter
Child Questionnaire	Child-age 10 years; annually thereafter
TEDDY book extraction	Every clinic visit

a Clinic visits are conducted every 3 months until 48 months and then every 6 months until 15 years. The children who developed islet autoimmunity continued a quarterly schedule until the diagnosis of diabetes or 15 years, whichever came first.

### 2.2 TEDDY Retention

The TEDDY study defined retention as the number of participants enrolled at any given point in time divided by the number still eligible to be followed at that time. Those diagnosed with type 1 diabetes or who died were removed from both the numerator and denominator. When participants actively withdrew from the study, the study staff collected the reason for withdrawal, contact information and permission for future contact. Passive withdrawals were those participants who did not respond to engagement attempts or had not completed any protocol elements over an extended period, but whose contact information was accurate. Both active and passive withdrawals were re-contacted annually and permitted to rejoin the study. Participants classified as lost-to-follow-up were disengaged from TEDDY with no accurate contact information.

**Fig. 1:**
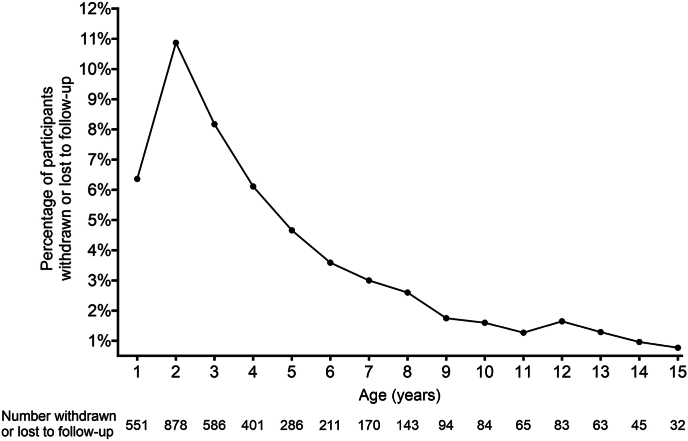
Percentage of children withdrawn or lost-to-follow-up by the age of the child.

As of September 30, 2023, 5633 (65 %) of the 8667 participants enrolled in TEDDY were still participating in the study. Fig. 1 describes the percentage of children withdrawn or lost-to-follow-up by the age of the child. The drop out was highest (11 %, *n* = 878) at 2 years of age, with the percentage of children dropping out declining thereafter to rates of less than 2 % from age 9 until 15. Among those who actively withdrew from the study, the most common reasons given included distress over the blood draw, the demanding nature of the protocol, and the family being too busy or experiencing stress. Among the 2634 withdrawn, 660 (25 %) participants later re-enrolled in the study.

### 2.3 Retention Strategies Classification

Based on an extensive systematic review of retention strategies among cohort studies conducted over the last decade, Teague et al. classified retention strategies into four broad categories: barrier-reduction, community-building, follow-up/reminder, and tracing strategies [1]. For this report, study coordinators from the six clinical centers compiled a list of all implemented retention strategies based on reviewing study documentation, retention presentations, and meeting minutes since the initiation of the TEDDY study. Two coordinators independently classified each TEDDY study strategy into one of the four Teague categories. The two independent coding exercises were compared, and the rare discordant results were discussed and reconciled. Several TEDDY retention strategies did not fit into one of the four Teague et al. categories. Consequently, a fifth category of strategies unique to TEDDY was added to the classification system.

## 3 Results

### 3.1 Retention Strategy Implementation

A summary of the retention strategies employed by the TEDDY study is described in Table 2A–D. For each Teague domain and individual strategy, specific examples used in the TEDDY Study are described. Table 3 describes the strategies unique to the TEDDY Study.

**Table 2A-D:** Mapping of TEDDY Retention Strategies used 2004–2020 to Meta-Analysis Typology of 4 Strategy Domains and 144 Individual Strategies Developed by Teague et al. [1].

A. DOMAIN: BARRIER REDUCTION With Individual Strategy Listed	EXAMPLES USED IN TEDDY
Adapt materials for different languages	*English, Finish, German, Swedish, Spanish added in one US site*
Adjust lab to be more home-like, less clinical	*Age-appropriate child themed décor, playroom/toys*
Assistance with postage costs	*Pre-paid postal and courier for returning questionnaires and lab samples*
Assistance with transport, parking, directions	*Where needed: taxi, ride services, bus fare, parking*
Catering/refreshments	*Standard office refreshments (*e.g. *Coffee, snacks); lite meal after Oral Glucose Tolerance Test*
Consistency in research staff	*Center-specific approaches included: 1) Assigned clinician as single point of contact for data collection, scheduling, results communication; 2) Participant able to request specific clinician*
Extended data collection window	*Changes in data collection frequency -e.g. stool samples from monthly to quarterly,*
Flexibility of research team (e.g., hours called, scheduling)	*Flexible clinic visit times (evenings, weekends)*
Hiring, training, and support of staff	*Centralized training meetings, local training, opportunities for professional development, focus on team development and staff retention*
Matching staff to participants, e.g., by language spoken, nature of questions	*Bilingual (Spanish/English) staff at 1 center that recruited Spanish speaking participants*
Prioritizing measures	*Prioritized endpoint data (blood for autoantibody assay) and exposure data (TEDDY Book) when doing tailored protocol, Blood draw only visits*
Recruiting for long-term retention	*Video describing study used for recruitment and informed consent and re-enrollment*
Simple, efficient procedure	*Combined phone and in-person to make clinic time shorter, more efficient; Abbreviated diet data collection*
Site and home visits	*Home visits, off-site clinics at locations closer to participant's home, mobile vans, long distance protocol for families who moved away from the clinic area.*
Skip waves	*Protocol Flexibility: Tailored to individual needs when necessary; skipping collection of selected items*
Splitting data collection over multiple sessions	*Used asynchronistic data collection for parent and child*
Toll-free project phone number	*Call routing from centralized number to study smart phones*
**Barrier-Reduction Strategies not used in TEDDY:** Survey design, Schedule two participants simultaneously, pilot testing, minimizing time between data collection points, focus group on survey design, partial data collected from proxy, anonymity for participants, childcare, advisory group, adjusted inclusion criteria	

A. DOMAIN: COMMUNITY BUILDING With Individual Strategy Listed	EXAMPLES USED IN TEDDY
Branding	*TEDDY logo used on all materials* e.g. *presents, questionnaires, brochures, labels*
Certificate of appreciation/completion	*Completion of specific protocol items*, *Certificate recognizing the child's halfway point in study at age 7.5 years and at study completion at 15 years.*
Educating the community on research	*Social media, blogs, study-wide and local clinical center websites. Publications in newspapers. Brochures. Talks at public meetings.*
Emphasizing the benefits of study	*Knowledge about T1D, symptoms and child's T1D risk; benefits of early diagnosis; lower risk of ketoacidosis; possibility to participate in prevention studies.*
Events/opportunity to meet other participants	*Science days, museum events, park days, parent/family evening events for study updates and meeting investigators.*
Gift/freebies	*Birthday presents; gift after blood draw or other protocol items.*
Hiring, training, and support of staff	*Both center specific training and support, study-wide training meetings for all staff members, regular conference calls*
Letter from chief investigator	*Center specific newsletters yearly to families*
Media coverage	*TV, magazine, and news articles that highlighted the TEDDY Study*
Newsletter/e-newsletter	*Newsletters sent to both parents and children separately and center specific.*
Opportunity to participate in other research	*Clinical Trials/Prevention studies for T1D*
Photo album	*Photos taken at study visit and shared with participant.*
Building rapport	*Conversation log to document the personal events and family milestones outside of study visit to build connection for future interactions.*
Sharing study results	*Results shared on websites, newspapers, social media, newsletters, published article links and lay summaries*
Social media	*Center-specific Facebook page, YouTube, Blogs*
Thank you, birthday, and holiday cards	*Same TEDDY birthday present and holiday cards for all children, center specific birthday and thank you cards.*
Time with chief investigator	*Parents evening events, research doctor available for participants*
Website	*Study-wide and center specific websites to inform participants and families.*
***Community Building Strategies not used in TEDDY:*** Champion participants, Gaining support of relevant institutions and organizations, Study membership card	

A. DOMAIN: FOLLOW-UP/REMINDER STRATEGIES With Individual Strategy Listed	EXAMPLES USED IN TEDDY
Follow-up brochure	*Materials developed and shared to inform parents of Follow Up study*
Budgeting for multiple contact attempts	*Use of a variety of contact approaches in scheduling and re-engagement protocol*
Extra incentive to complete all data collection points	*US Centers only: variable payments based on completed data items (stool samples and diet records)*
Gift/freebies incentives (e.g., t-shirts, discount cards)	*Birthday presents; gift after completing blood draw or other protocol items; coupons*
Hiring, training, and support of staff	*Centralized training meetings, local training, opportunities for professional development, focus on team development and staff retention*
Incentive (cash/vouchers)	*US centers only paid participants for completion of different elements of the study protocol*
Incentives raffles/competitions	*Select centers implemented raffles to increase stool sample compliance*
Increased incentive for hard-to-reach	*Some US Centers used additional pay for completing study visit after extended time of not attending*
Limiting number of calls etc. based on participants' response	*Contact attempts were limited to those who did not respond*
Medical assistance (e.g., diagnostic testing)	*Results from blood draw and were shared with participant after each visit (*e.g.*, type 1 diabetes, celiac and thyroid disease autoantibodies)*
Phone Follow-up	*Phone calls to discuss positive results*
Provide referrals, e.g., medical or legal	*Referral to specialist for needle phobia/referral to psychologist based on questionnaire response to psychosocial measures*
Email reminder	*Email used for scheduling, study follow up, reminder of visit*
Phone call reminder	*Phone used for scheduling, study follow up, reminder of visit*
Postcard/letter reminder	*Letter sent annually to inactive or withdrawn participants*
SMS reminder	*SMS (text) used for scheduling, study follow up, reminder of visit*
Reminders (unspecified)	*Sent reminders for specific data items multiple times*
**Follow-up/Reminder Strategies not used in TEDDY:** Incentive increasing value over time; Resend survey once, Resend survey multiple times; SMS follow-up; Website follow-up; Face-to-face reminder (e.g., home visit)	

D. DOMAIN: TRACING STRATEGIES With Individual Strategy Listed	EXAMPLES USED IN TEDDY
Tracing via alternative contacts	*Alternate contact information collected and reviewed at each study visit*
Case-review meetings	*Team review of families challenged by completing study protocol.*
Tracing via change of address cards	*Used return mail option to get a forwarding address for participants who moved.*
Tracing via email	*Attempted to gather multiple emails for household to use to locate*
Hiring, training, and support of staff	*Local staff trained on specific contact approaches*
Tracing via letter	*Letters sent out with return address request to get updated address*
Tracing via phone call	*Multiple phone numbers collected and used to contact*
Tracing via public records	*Use EU registries/online directory (*whitepages.com*) to update address*
Tracing via tracking database	*Each clinical center used locally designed tracking database*
Tracing via update your details form	*Use of TEDDY Update Form in re-engagement protocol so participants would share current contact information*
**Tracing Strategies not used in TEDDY:** Extensive location tracing information, Tracing via house visit, incentive for staff members, incentive to update contact details, locator form documentation, private investigator, SMS, social media, website, non-public records

**Table 3:** Retention strategies unique to TEDDY with specific implementation examples.

UNIQUE TEDDY STRATEGIES	EXAMPLES OF SPECIFIC IMPLEMENTATION
Study structures to support retention	*Coordinator/retention calls, study staff training, Collaboration with Psychosocial Committee (surveys, analysis to guide strategy development) Child Engagement Committee, local retention coordinators*
Risk Communication/Education Strategies	*Annual risk conversation, use of pictographs to illustrate risk, Junior Scientist books, child focused informational videos to explain autoimmunity and diabetes.*
Data-informed retention strategies	*Study wide parent survey, parent feedback cards, child feedback card using happy to sad faces scale. Implementation of High Risk for Early Withdrawal (HREW) with tailored intervention, Retention-Compliance Score (RCS) Report to identify and address challenging protocol elements, Enrollment Status Report (ESR)*
Re-engagement protocol	*Yearly mailed retention materials to re-engage inactive and withdrawn participants, offer of a one-time blood draw.*

#### 3.1.1 Teague domain: Barrier-reduction strategies

Barrier-reduction strategies included efforts to help participants meet the study protocol. These included flexibility in scheduling or location of visits, adapting materials to all relevant languages, providing childcare or transportation assistance, and intermittent negotiations in data collection while maintaining fidelity to the protocol (Table 2A).

All centers considered the personal relationship between study staff and participants important. However, TEDDY centers were organized in a way suited to the specific conditions and constraints of their environment. The professional backgrounds of the staff varied significantly by country and center. Some TEDDY centers had multiple staff members managing scheduling and conducting research visits. Other centers opted for a case management approach with families assigned to one dedicated staff member who followed the family for all visits, and were responsible for scheduling, the blood draw, and the data collection. An analysis of case management approach found it to be particularly effective in Europe but less commonly used in the US [13]. However, over time, many of the centers gravitated towards a hybrid case-management approach where there was a consistent team or individual in contact with the TEDDY participant.

#### 3.1.2 Teague domain: community-building strategies

Community-building strategies included such things as creating a study logo, gifts with the study logo, and study newsletters (Table 2B). The greatest number of TEDDY retention strategies fall in this domain. A TEDDY logo was used in all study communications, presentations, and annual birthday gifts. As the study participants approached school age, the focus of retention efforts moved away from being exclusively parent to family-child focused engagement strategies. Many sites arranged science-focused events where TEDDY participants could meet each other as well as the TEDDY investigators. Children were given the opportunity to have a pen-pal from another TEDDY clinic. YouTube videos were created and shown at clinic visits, distributed through newsletters, and posted on social media that emphasized the larger TEDDY community. The TEDDY Around the World video, featuring actual TEDDY children, gave a snapshot of each clinical center where the children lived and activities they might do for fun [14]. The goal was to emphasize the global span of TEDDY and show children how they were part of something special. Furthermore, it sparked their imagination that TEDDY is much bigger than what they saw at their study visits.

#### 3.1.3 Teague domain: Follow up/reminder strategies

This domain included strategies to encourage participant compliance with study visits and typically involved incentives and various forms of reminders (Table 2C). There were differences between the US and EU human subjects’ regulations regarding the use of cash incentives. Only the US centers used cash payments. Gifts, gift cards, vouchers, and other kinds of non-cash support were used more broadly, with variability based on local human subject review board considerations.

TEDDY used multiple forms of communication to keep families engaged by text, email, letters, and phone calls. Keeping up with the family's mode of contact preferences and utilizing technology effectively required each center to be both technologically up-to-date and creative.

#### 3.1.4 Teague domain: tracing strategies

Tracing strategies involved collecting detailed contact information so the participant could be located even after long periods of absence from the study protocol (Table 2D). In the TEDDY study, the contact information for all primary caregivers and alternate contacts outside of the home was updated at each study visit. The European centers had the additional advantage of country-specific unified registration systems to enable better tracking of participants.

#### 3.1.5 Unique TEDDY strategies

These strategies go beyond what has been described in the literature and include study structures to support retention, risk communication and education strategies, data-informed retention strategies, and the re-engagement protocol (Table 3).

***Study structures to*** support ***retention*** included the designated role of the TEDDY Study Coordinator Committee (SCC) in the monitoring of study compliance and retention as well as the design and implementation of retention strategies. The SCC members focused specifically on the participant's experience, providing important observations related to participant's needs and concerns that were the foundation for successful retention. The collaboration between the SCC and the TEDDY Psychosocial Committee resulted in the formation of the Child Engagement Committee which developed age-appropriate strategies for keeping child participants engaged and informed about the study. The TEDDY Psychosocial Committee conducted parent surveys and identified factors collected at enrollment that predicted study drop-out that were then used to guide retention strategies.

***Risk communication and education strategies*** focused on the purpose of the TEDDY study and the TEDDY child's risk for type 1 diabetes*.* The study coordinators developed and used a variety of methods, including pictographs, for communicating and assessing the understanding of risk on an ongoing basis. This reinforced the importance of study participation for both the parent and child.

The Child Engagement Committee created a series of storybooks to help children understand the TEDDY study which were translated into all five TEDDY languages. The first small picture book, “We Go To TEDDY,” was given to families of 2–3-year-old children [15]. The book used colorful pictures and simple sentences to explain how the character Willie participated in his TEDDY visit. A second storybook and accompanying activity book continued with the story of Will and introduced his classmate Emma, who was also a TEDDY participant. It was developed for school-aged children and was designed to be read together with an adult [16]. The book further explained type 1 diabetes, why children were in TEDDY, and how scientists studied the many samples collected in the study. The book referenced a “Junior Scientist” pin that was given to all study participants. At age 10, children were given a 40-page chapter book further explaining the development of type 1 diabetes and a TEDDY child's risk for developing the disease [17]. The same characters created continuity and community, while helping children understand their role in the TEDDY study. All of the TEDDY books are available online through the NIDDK Central Repository [18].

The Child Engagement Committee also developed a series of videos that educate both parents and children about how the study related to them directly. One video followed the journey of a blood sample from the TEDDY clinic to the study laboratory for processing, and the travel to the large NIH repository for storage [19]. Another series of videos used illustrations and text to explain diabetes autoimmunity [20].

***Data-informed retention strategies*** used study data to identify targets for the development of specific retention strategies.

*Using Participant Feedback to Guide Retention Strategies*. Early in TEDDY, parents were surveyed as to their reasons for declining TEDDY enrollment [21] and their reasons for leaving TEDDY [22]. The blood draw in an infant was a common reason for both failure to enroll and withdrawing from the study. Consequently, in-service training was provided to all TEDDY staff on age-appropriate preparation and distraction methods to reduce child anxiety and distress. Starting at age 5 years, children were asked to rate their blood draw experience using smiley faces or stars, which helped the study staff individualize this blood draw experience to the needs of each child.

*Identifying Those at High Risk for Study Withdrawal*. Using data collected in the first two years, an analysis was conducted to identify factors assessed at study enrollment that were associated with dropping out of TEDDY in the first year [23]. Based on these findings, a risk score was developed to identify those who were at high risk for study drop out. All newly enrolled study participants had a risk score calculated, and study coordinators were informed of those who scored as high risk for drop out. The coordinators then designed a tailored intervention specifically for each high-risk family, leading to improvement in study retention [24]. Tailored interventions concentrated on the most challenging aspects of the protocol for that particularly family. For example, a single mother might have needed help with childcare for her other children or flexible study scheduling to accommodate her work schedule. A home capillary collection protocol was created as an option for families who could not attend their visits during the scheduled clinic times.

*Retention-Compliance Score (RCS) Report.* The RCS score was based on each participant's compliance with elements of the study protocol and was calculated periodically for all enrolled participants. This aided the study coordinator to identify those in need of specific targeted interventions. For example, participants with a low RCS score often had poor compliance with the stool sample collection, the 3-day food record and the activity meter for which specific incentives and age-appropriate information materials could be given (e.g., print outs of each child's activity from the meter they wore). These were designed to increase the family's motivation to comply with these aspects of the study protocol.

In the first 10 years of the TEDDY Study, all questionnaires were in paper format, requiring additional mailings prior to the study visit. Families provided feedback that remembering to bring the forms to the clinic visit and the lengthened time at the clinic visit to do them in-person was difficult. To reduce this study burden, the TEDDY Data Coordinating Center created an online participant portal where parents, and eventually children, completed the questionnaires on their personal computer or mobile device. The portal had the advantage of shortening clinic visit time and reducing the staff burden for data entry and follow-up of missing forms.

*The Enrollment Status Report (ESR).* This report permitted the study staff to determine the enrollment status (enrolled, developed type 1 diabetes, withdrawn, lost to follow-up, died, and rejoined) of any participant at any time and among the enrolled, the degree of activity in the previous 2 years for that individual. The report also provided data for the total cohort, each clinical center, and the site/locations within the center, serving to alert centers to changes in withdrawal patterns and level of engagement.

***TEDDY's Re-engagement protocol*** provided a systematic approach for participants to rejoin the study after a period of withdrawal. All withdrawn participants who agreed to future contact were contacted yearly to assess whether the child had developed type 1 diabetes and if not, to specifically invite families to resume their study participation. Study staff used this approach to communicate that life circumstances, family dynamics, and the TEDDY child's stage of development could have changed, allowing study participation to become possible again. A robust tracing protocol, as noted above, was critical to the success of this strategy. Approximately 25 % of those who withdrew from the TEDDY study later rejoined.

## 4 Discussion

Since 2003, the TEDDY Study has been a major investment of the National Institutes of Health to establish an international cohort of children at high genetic risk for developing type 1 diabetes and to follow them from birth to 15 years to identify environmental triggers of this disease. Retention of these children in a demanding protocol was clearly a challenge. TEDDY employed over 100 retention strategies; many were consistent with domains identified by Teague et al. in their comprehensive review: barrier reduction strategies, community building strategies, follow-up/reminder strategies, and tracing strategies [1]. Although all were useful, the community building strategies seemed particularly effective. Children and families liked to see themselves as part of a larger effort. In a survey asking why parents stayed in TEDDY, “helping science discover cause of diabetes” was a common reason given [25].

TEDDY retention rates were not uniform across time. The highest rate of withdrawal (11 %) was during the first two years of participation. Parents reported that the blood draw in infants and children less than 2 years was a major concern. Parents also identified the demanding protocol as the main reason for leaving the study during this stressful stage of childhood [22]. During the enrollment phase of the TEDDY Study, study staff had to split their time between enrolling new participants and retaining those enrolled which may have further contributed to the high drop-out rates in the first two years after enrollment. As children reached school age, the rate of withdrawal fell from 5 % to 3 %. By this point, most participants were invested in the research and the coordinator's focus on retention became a priority. This is evidenced by the rate of withdrawal continuing to decline to less than 2 % from the ages of 9–15 years.

There is no question that the relationship between study staff and the participant's family was critical to study retention. Early in the study, survey responses showed that having someone watching their child for development of type 1 diabetes was the most important reason for staying in the study [25]. Fifteen years later, parents identified this same reason as the best part of the study. There was some evidence that having a particular study staff member assigned to a family was particularly effective in Europe although it was used less often in the US [13]. However, by the end of the TEDDY study, most sites had gravitated toward some form of a case management approach.

In a longitudinal pediatric study, the child's changing developmental level plays a critical role. Not only were concerns about the blood-draw in infants and very young children a source of higher drop-out rates, but the information provided had to be changed as the child grew older and reached the age of assent. TEDDY concentrated a great deal of time, energy, and resources on ensuring the children were well informed about what was involved with their study participation and its importance. The Child Engagement Committee was charged with creating suitable child content and implementing strategies focused on the child. Age-appropriate books were developed and translated into all TEDDY languages. Incentives and community-based activities changed as the child grew older. Children's feedback about TEDDY procedures was collected and used as the child grew older. The ability of any protocol to adapt to the child's developmental level is critical to its success.

One of the unique contributions of TEDDY is its development of data-informed retention strategies. Parents were surveyed about their experiences in TEDDY and their concerns guided changes to the protocol and specific intervention strategies – the efforts to develop methods to do the blood draw in very young children with minimal distress being one of the most utilized examples. Factors identified at study enrollment that were associated with study dropout in the first year were used to develop a risk score, permitting study staff to focus their efforts on those most likely to leave TEDDY, with good success [12,14]. The development of a Retention-Compliance Score and an Enrollment Status Report that could be accessed in real-time, permitted study staff to focus effort on those most likely to drop out or those aspects of the study protocol most challenging for participants to complete.

Recognizing there were many reasons for withdrawal associated with family characteristics not in the study's control resulted in the TEDDY coordinators instituting the re-enrollment protocol. This protocol was an important strategy for long-term engagement and data completeness. In addition to assessing type 1 diabetes status in the child who had withdrawn from TEDDY, the re-engagement protocol focused on inviting families back at any time. In a long-term study, having the ability to check back in after several years when family conditions may have changed, and the child is older can create a pathway to rejoining. The re-engagement protocol required extensive staff resources, but it resulted in 25 % of those who withdrew re-joining the study.

TEDDY's structure was also crucial to its success. The SCC played a critical role in monitoring study retention and compliance and developing retention strategies in response. The Psychosocial Committee conducted the parent surveys and analyses that identified targets to reduce drop-out. The Child Engagement Committee played a critical role in developing age-appropriate materials and retention strategies. TEDDY devoted resources to these efforts on an ongoing basis.

Although TEDDY put considerable resources into study retention, multiple strategies were used with no plan to evaluate which ones were most effective or cost-effective. While Robinson suggests studies should employ multiple strategies to best improve retention [26], it was difficult to accurately measure the effectiveness of any one strategy at a given point. Fortunately, it was apparent that the dropout of participants continued to decline once the retention strategies were consistently implemented.

## 5 Conclusion

Whether a clinical trial or a longitudinal observational study, cohort retention is critical to the validity and generalizability of the research findings. Observational studies need to be particularly attentive to the threats arising from attrition and the resulting missing data. The TEDDY study experience highlights the importance of using multiple retention strategies. Those that seemed particularly effective included creating a sense of community, the use of data collected in real time to identify targets for interventions, and the implementation of a re-enrollment protocol that permitted those who withdrew to return to the study. The creation of structure and designated resources should be taken into account in the early phases of start-up and considered throughout all stages of the study.

## CRediT authorship contribution statement

**Patricia Gesualdo:** Writing – review & editing, Writing – original draft, Visualization, Supervision, Project administration, Methodology, Investigation, Data curation, Conceptualization. **Jessica Melin:** Writing – original draft, Visualization, Project administration, Methodology, Investigation, Data curation, Conceptualization. **Rachel Karban:** Writing – review & editing, Writing – original draft, Validation, Methodology, Formal analysis. **Claire Crouch:** Writing – review & editing, Writing – original draft, Validation, Methodology, Investigation, Formal analysis. **Michael Killian:** Writing – review & editing, Methodology, Investigation, Conceptualization. **Diane Hopkins:** Writing – review & editing, Resources, Methodology, Investigation. **Annika Adamsson:** Writing – review & editing, Resources, Methodology, Investigation. **Joanna Stock:** Writing – review & editing, Resources, Methodology, Investigation. **Suzanne Bennett Johnson:** Writing – review & editing, Writing – original draft, Supervision, Resources, Investigation, Conceptualization. **Judith Baxter:** Writing – review & editing, Writing – original draft, Supervision, Resources, Project administration, Methodology, Investigation, Data curation, Conceptualization.

## Ethics approval and consent to participate

The TEDDY study was approved by local U.S. Institutional Review Boards and European Ethics Committee Boards in Colorado's Colorado Multiple Institutional Review Board 04–0361, Georgia's Medical College of Georgia Human Assurance Committee (2004–2010), Georgia Health Sciences University Human Assurance Committee (2011–2012), Georgia Regents University Institutional Review Board (2013–2016), Augusta University Institutional Review Board (2017-present) HAC 0405380, Florida's University of Florida Health Center Institutional Review Board IRB201600277, Washington state's Washington State Institutional Review Board (2004–2012) and Western Institutional Review Board (2013–2019), WCG IRB (2020-present) 20130211, Finland's Ethics Committee of the Hospital District of Southwest Finland Dnro168/2004, Germany's Bayerischen Landesärztekammer (Bavarian Medical Association) Ethics Committee 04089, Sweden's Regional Ethics Board in Lund, Section 2 (2004–2012) and Lund University Committee for Continuing Ethical Review (2013–2021), Swedish Ethical Review Authority (2022-present) 217/2004.

## Consent for publication

Not applicable.

## Availability of data and materials

Data and retention materials from The Environmental Determinants of Diabetes in the Young (https://doi.org/10.58020/y3jk-x087. Click or tap if you trust this link.">https://doi.org/10.58020/y3jk-x087) reported here will be made available for request at the NIDDK Central Repository (NIDDK-CR) website, Resources for Research (R4R), https://repository.niddk.nih.gov/. Click or tap if you trust this link.">https://repository.niddk.nih.gov/

## Funding

The TEDDY Study is funded by U01 DK63829, U01 DK63861, U01 DK63821, U01 DK63865, U01 DK63863, U01 DK63836, U01 DK63790, UC4 DK63829, UC4 DK63861, UC4 DK63821, UC4 DK63865, UC4 DK63863, UC4 DK63836, UC4 DK95300, UC4 DK100238, UC4 DK106955, UC4 DK112243, UC4 DK117483, U01 DK124166, U01 DK128847, and Contract No. HHSN267200700014C from the National Institute of Diabetes and Digestive and Kidney Diseases (NIDDK), National Institute of Allergy and Infectious Diseases (NIAID), Eunice Kennedy Shriver National Institute of Child Health and Human Development (NICHD), National Institute of Environmental Health Sciences (NIEHS), Centers for Disease Control and Prevention (CDC), and JDRF. This work is supported in part by the 10.13039/100000002NIH/10.13039/100006108NCATS Clinical and Translational Science Awards to the 10.13039/100007698University of Florida (UL1 TR000064) and the 10.13039/100010174University of Colorado (UL1 TR002535). The content is solely the responsibility of the authors and does not necessarily represent the official views of the National Institutes of Health.

## Declaration of competing interest

The authors declare that they have no known competing financial interests or personal relationships that could have appeared to influence the work reported in this paper.

## Data Availability

A statement of the availability of data and materials is noted on page 19

## Abbreviations:

ESR: Enrollment Status Report
HREW: High Risk for Early Withdrawal
NIH: National Institutes of Health
RCS: Retention-Compliance Score Report
SCC: Study Coordinator Committee
T1D: Type 1 Diabetes
TEDDY: The Environmental Determinants of Diabetes in the Young
